# Performance characteristics of ^18^F–fluorodeoxyglucose in non-infected hip replacement

**DOI:** 10.3389/fmed.2022.1043812

**Published:** 2023-01-05

**Authors:** Yiqun Wang, Lulu Yuan, Yinqiao Du, Honghong Liu, Qingxiao Li, Yan Chang, Yuanyuan Shi, Yanmei Wang, Xiaolin Meng, Yonggang Zhou, Shulin Yao, Jiahe Tian

**Affiliations:** ^1^Department of Nuclear Medicine, The First Medical Centre, Chinese People’s Liberation Army (PLA) General Hospital, Beijing, China; ^2^Beijing Key Laboratory of Sports Injuries, Department of Sports Medicine, Institute of Sports Medicine of Peking University, Peking University Third Hospital, Beijing, China; ^3^Department of Orthopedics Surgery, The First Medical Center, Chinese People’s Liberation Army (PLA) General Hospital, Beijing, China; ^4^Department of Orthopedics Surgery, The Fourth Medical Centre, Chinese People’s Liberation Army (PLA) General Hospital, Beijing, China; ^5^General Electric (GE) Healthcare China, Shanghai, China

**Keywords:** ^18^F-FDG, PET/CT, hip, arthroplasty, image analysis

## Abstract

**Purpose:**

The aim of this study was to retrospectively analyze 18F-fluorodeoxyglucose (^18^F-FDG) positron emission tomography (PET)/ computed tomography (CT) images of non-infected hip arthroplasty patients and summarize findings that may be useful for clinical practice.

**Methods:**

^18^F-FDG PET/CT images of non-infected hip arthroplasty patients were collected from September 2009 to August 2021. The region of interest was independently delineated by two physicians and maximum standardized uptake values (SUV_max_) were recorded and compared. Serologic data were also collected and the correlation between SUV_max_ and serologic parameters was analyzed, while the images were classified based on the ^18^F-FDG uptake pattern in the images using the diagnostic criteria proposed by Reinartz et al. (9). The interval between hip replacement and PET/CT was classified by year and the characteristics of the two groups were compared. The images of patients who underwent PET/CT multiple times were analyzed dynamically.

**Results:**

A total of 121 examinations were included; six patients underwent PET/CT twice and two patients had three scans. There were no significant correlations between SUV_max_ and serologic results. The interobserver agreement between the two physicians in the classification according to the criteria of Reinartz et al. (9) was 0.957 (*P* < 0.005). Although there was non-specific uptake in cases with an arthroplasty-to-PET/CT interval this was non-significant. Additionally, ^18^F-FDG showed potential utility for dynamic observation of the condition of the hip.

**Conclusion:**

SUV_max_ provided information independent of serologic results, meanwhile ^18^F-FDG showed potential applicability to the dynamic monitoring of hip arthroplasty-related diseases. However, the presence of blood vessels and muscles affected image interpretation and the specificity of ^18^F-FDG was not optimal. A more specific radionuclide is needed to maximize the benefits of using PET/CT for the assessment of periprosthetic joint infection (PJI).

## 1. Introduction

^18^F-fluorodeoxyglucose (FDG) is widely used in precision medicine mostly in the field of cancer, and is increasingly being applied to other diseases including hip periprosthetic joint infection (PJI). ^18^F-FDG was first applied to PJI in the 20th century (1–3) and its use peaked in the following years (4–13) but has declined in the last 5 years (14, 15), raising questions as to the utility of ^18^F-FDG in the diagnosis of hip PJI.

**FIGURE 1:**
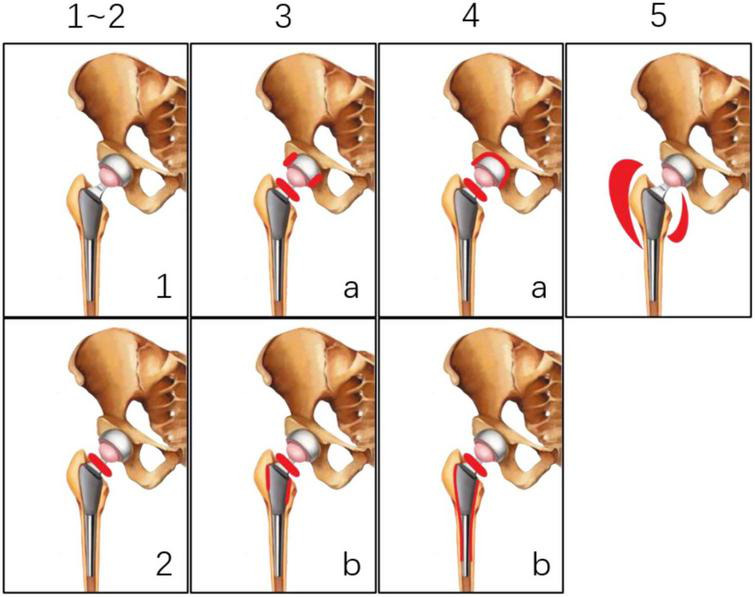
Classification system proposed by Reinartz et al. (9).

Zhuang et al. (2) proposed increased uptake at the bone–prosthesis interface as a diagnostic criterion for PJI, without a quantifiable indicator, such as a cutoff value for the maximum standardized uptake value (SUV_max_). The sensitivity, specificity, and accuracy in their report were 90, 89.3, and 89.5%, respectively. Vanquickenborne et al. (16) subsequently defined infection as uptake around the implant greater than that in the contralateral region, which had sensitivity, specificity, and accuracy of 87.5, 77.8, and 82.4%, respectively. Although loosening of the artificial hip joint is associated with inflammation that can increase glycolysis, it is unclear how this can be differentiated from PJI in ^18^F-FDG positron emission tomography (PET)/computed tomography (CT) images. With a greater understanding of this phenomenon, it was proposed that the location of ^18^F-FDG uptake was more relevant than the intensity (17). In 2005, Reinartz et al. (9) proposed a classification system to diagnose PJI based on uptake patterns in ^18^F-FDG PET/CT images (Figure 1) that yielded highly satisfactory results, with sensitivity, specificity, and accuracy of 0.94, 0.95, and 0.95, respectively. These diagnostic criteria were significant not because of their accuracy but because as a molecular imaging modality, PET/CT has the unique advantages of high sensitivity and the possibility of visualizing the exact location of a lesion and is therefore far more useful than a serologic cutoff value. Some investigators have since diagnosed PJI or proposed ^18^F-NaF uptake patterns based on this classification (11, 18, 19). While the results were acceptable, there are few relevant reports and cases in the literature, and infections between the acetabulum or femur and the prosthesis were not included in this classification.

Most studies to date on ^18^F-FDG and hip arthroplasty have focused on the diagnosis of infection, and there have been few reports on the characteristics, advantages, and disadvantages of ^18^F-FDG after hip replacement; moreover, it is not known what additional information ^18^F-FDG PET/CT can provide compared to other examinations that can help orthopedists diagnose PJI. To address these issues, the present study retrospectively analyzed ^18^F-FDG PET/CT images of non-infected hip arthroplasty patients treated at our center with the aim of summarizing clinically useful findings.

## 2. Materials and methods

### 2.1. Study sample

Patients with hip arthroplasty who were referred for PET/CT at the Chinese People’s Liberation Army General Hospital from September 2009 to March 2021 were included in this study without a limit to the type of prostheses. Exclusion criteria were as follows: unable to obtain a valid image; non-^18^F-FDG radionuclide; age <18 years; and cases of intramedullary nail fixation. Serologic test results were collected at the same time and the final diagnosis was determined at a follow-up of at least 1 year using 2011 Musculoskeletal Infection Society (MSIS) criteria. Written, informed consent forms and permission to use patient data for scientific purposes were obtained at the time of the patient’s examination. Because of the retrospective nature of this study, a waiver was granted by the institutional review board of Chinese People’s Liberation Army General Hospital and no additional informed consent was needed.

### 2.2. PET/CT examination

All patients were scanned using an integrated whole-body PET/CT scanner (uMI510, United Imaging Healthcare, Shanghai, China; or Discovery 710, GE Healthcare, Chicago, IL, USA) and had fasted for at least 6 h before the scan with plasma glucose levels <11.1 mmol/L and rested for at least 30 min in a quiet waiting room before ^18^F-FDG administration. The acquisition time was 150 s per bed position. Low-dose CT (110 kV, 25–50 mAs) was chosen for anatomic localization and attenuation correction. Images were reconstructed using a standard ordered-subset expectation-maximization algorithm. The patients were intravenously injected with ^18^F-FDG (Atomic High-Tech Co., Changzhou, China; radiochemical purity >95%); the radioactive dose was calculated according to body weight, with a dose range of 3.70–4.44 MBq/kg (0.01–0.12 mCi/kg). The PET/CT scan was performed 1 h after injection.

All PET images were transformed into SUV units by normalizing the activity concentration to the administered ^18^F-FDG dose and the patient’s body weight after decay correction. The images were reconstructed with CT attenuation correction using the ordered subset expectation maximization algorithm. All CT images were converted to a bone window.

### 2.3. PET/CT analysis

All PET/CT examinations were anonymized in this analysis. The images were independently evaluated by two experienced nuclear medicine physicians. Both physicians were blinded to clinical data and the final diagnosis.

To obtain SUV_max_, the region of interest, which was the area with abnormal uptake around the hip prosthesis, was independently delineated by the two physicians and SUV_max_ values were recorded and compared. Images were classified based on ^18^F-FDG uptake pattern according to Reinartz et al. (9) [(1)-no increase in ^18^F-FDG uptake; (2)-increased uptake of [^18^F]FDG in the area around the femoral neck; (3)-increased uptake of ^18^F-FDG in the femoral neck and parts of the prosthesis-bone interface of the acetabular cup and/or proximal stem; (4)-increased uptake of ^18^F-FDG in the femoral neck and whole prosthesis-bone interface of the acetabular cup and/or in the wide parts of the stem; (5)-uptake of ^18^F-FDG in the periprosthetic soft tissue], and the consistency of the results obtained by the two physicians was compared. It should be noted that in this study, type IV or V PJI was not indicative of loosening or infection, but simply described an uptake pattern in the image.

Given that there can be non-specific FDG uptake for 6 months to 1 year after hip replacement due to tissue remodeling (9, 14, 15, 20, 21), the images were grouped by year and the performance characteristics of the two groups were compared. Additionally, images of patients who had multiple PET/CT scans were examined for changes over time to evaluate the efficacy of PET/CT in dynamic monitoring.

### 2.4. Statistical analysis

The baseline characteristics of the patients were described as continuous and dichotomous data, and are presented as the mean ± SD unless otherwise stated. The κ statistic was used to assess interobserver agreement (22, 23). A two-sided unpaired *t*-test was used for two-group comparisons. Correlations between SUV_max_ and serologic tests were analyzed with Pearson’s rank correlation. For all analyses, *P* < 0.05 was considered statistically significant.

## 3. Results

### 3.1. Patient characteristics

**FIGURE 2:**
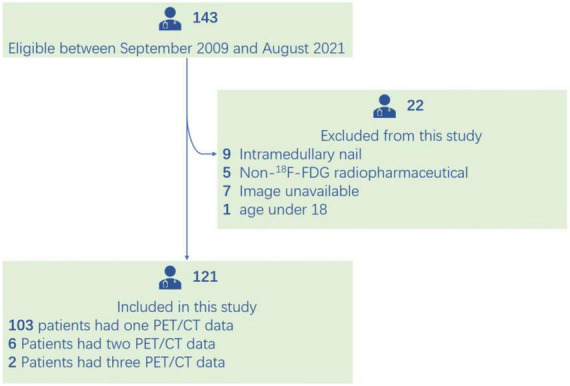
Flowchart of patient selection.

A total of 143 PET/CT images were identified between September 2009 and August 2021 and 22 were excluded. Of the including patients, six underwent two PET/CT scans and two underwent three scans (Figure 2). For ease of calculation and clarity of presentation, for patients underwent multiple scans, each scan was treated as an independent unit. The images were from 85 men and 36 women (mean age, 68.85 ± 12.80 years). The serologic examination included C-reactive protein (CRP), D-dimer, erythrocyte sedimentation rate, and interleukin six level. The mean interval between arthroplasty and PET/CT was 75.69 ± 56.75 months (range: 0.25–264.00 months). The patients’ clinical data are summarized in Table 1.

**TABLE 1:** Clinical data of included patients.

Parameter	Value
Age, years	67.85 ± 12.80 (28–98)
Sex, male	85
Body mass index, kg/m^2^	24.26 ± 3.63 (12.62–33.91)
**Purpose of PET/CT**		
Proximal femur tumor	5
Other tumors	81
Physical examination	14
PJI	4
Other	17
**Serologic test**		
CRP (*n* = 43)	2.79 ± 4.90 (0.05–18.82)
D-Dimer (*n* = 53)	1.39 ± 1.65 (0.29–9.39)
ESR (*n* = 20)	42.60 ± 31.22 (5.00–106.00)
IL-6 (*n* = 26)	43.52 ± 118.47 (2.00–613.90)
Time interval between arthroplasty and PET/CT, min	75.69 ± 56.75 (0.25–264.00)
SUV_max_	4.49 ± 3.63 (1.60–38.10)

CRP, C-reactive protein; CT, computed tomography; ESR, erythrocyte sedimentation rate; IL-6, interleukin 6; PET, positron emission tomography; PJI, periprosthetic joint infection; SUV_max_, maximum standardized uptake value.

### 3.2. ^18^F-FDG imaging performance

**FIGURE 3:**
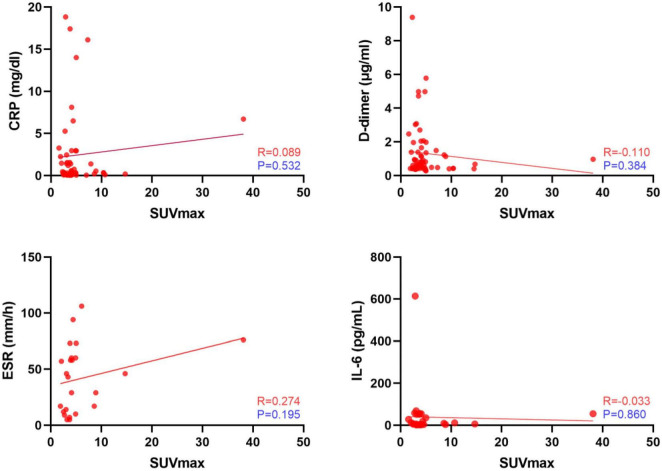
Linear regression analysis between standardized uptake values (SUV_max_) and serologic test results.

**FIGURE 4:**
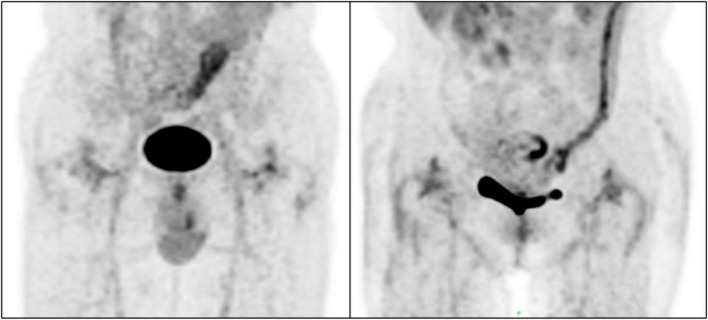
Non-specific uptake of ^18^F-fluorodeoxyglucose (^18^F-FDG) by blood vessels and muscles.

SUV_max_ ranged from 1.60 to 38.10 and the interobserver agreement was one. Linear regression analysis revealed no association between SUV_max_ and serologic parameters (Figure 3). Only four images were inconsistently classified by the two physicians according to the criteria of Reinartz et al. (9) (κ = 0.957, *P* < 0.005; Table 2). Blood vessels and muscles in the images appeared to influence image interpretation (Figure 4).

**TABLE 2:** Classification according to uptake pattern proposed by Reinartz et al. (9).

Classification	Reader 1	Reader 2
I	47	47
II/III	70	74
IV	5	5
V	23	19

**FIGURE 5:**
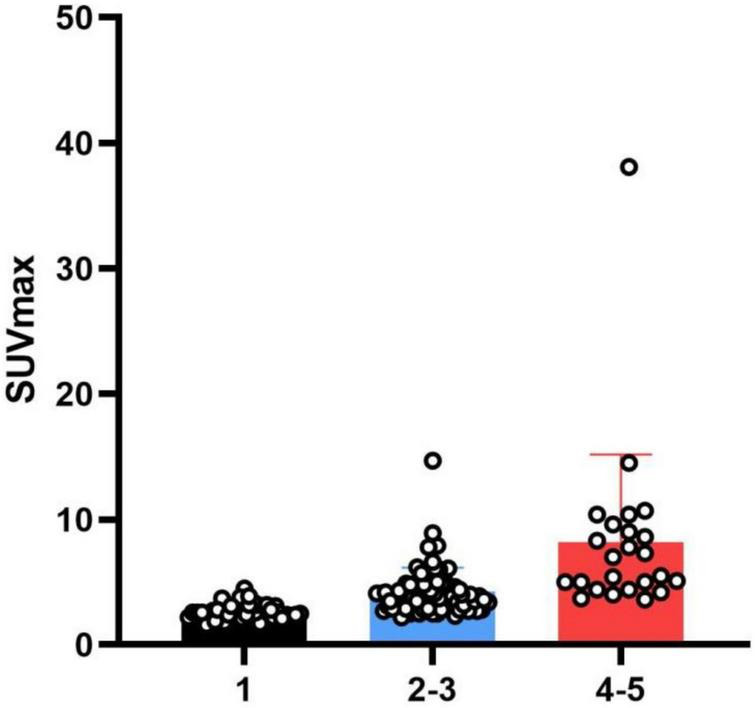
Comparison of standardized uptake values (SUV_max_) among different groups.

**FIGURE 6:**
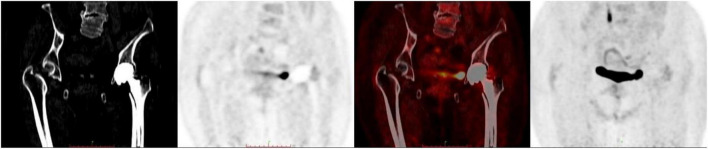
Case of interval time <6 months.

**FIGURE 7:**
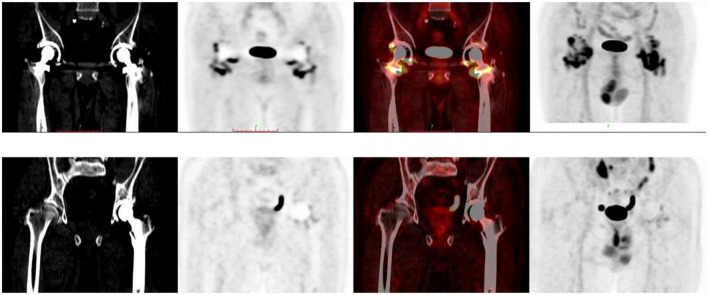
Different characteristics of wear cases.

**FIGURE 8:**
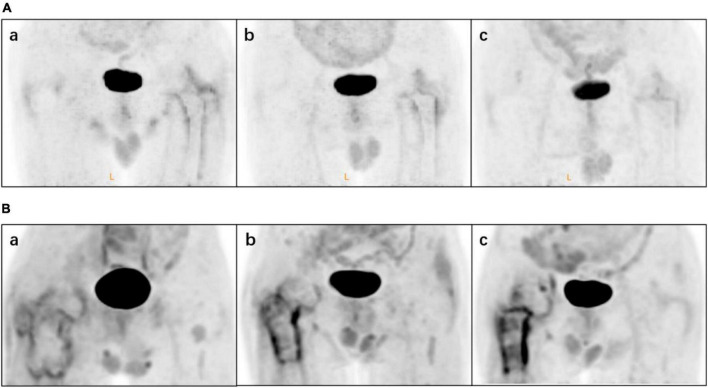
Two patients had multiple ^18^F-fluorodeoxyglucose (^18^F-FDG) positron emission tomography (PET)/computed tomography (CT) images. Patient **(A)** underwent total hip arthroplasty (THA) during chemotherapy: (a) 6 months, (b) 18 months and (c) 31 months postoperatively. Patient **(B)** with a tumor prosthesis underwent ^18^F-FDG at (a) 1 month, (b) 5 months and (c) 12 months postoperatively.

In the following analysis, classification by reader 2 was taken as the standard (through discussion, as for inconsistent results of analysis, the results of reader 2 were approved by both physicians). The SUV_max_ of types I, II/III, IV, and V differed significantly (Figure 5), suggesting that this value reflects differences in ^18^F-FDG uptake patterns. The images were classified according to the interval between arthroplasty and PET/CT (Table 3). For 20 images the interval was<1 year. For some patients who had just undergone surgery (interval <6 months), the images did not show diffuse uptake as expected (Figure 6), although the proportion of type V cases was larger than images obtained after 1 year (35 vs. 11.42%). For images obtained after 1 year, half of the images still showed non-specific uptake. Most of the prostheses showed wear, including some with significant uptake and some without (Figure 7). Among patients with multiple PET/CT scans, the images of two patients who each had three scans showed an unexpectedly high performance (Figure 8). One patient had a tumor prosthesis and the images showed a gradual increase in the intensity and range of ^18^F-FDG uptake; another patient had total hip arthroplasty and the interface between the femoral prosthesis and bone showed a gradual decrease in intensity and range.

**TABLE 3:** Classification according to the time interval.

	Interval	
**Classification**	**<1 year**	**≥1 year**
I	2	45
II/III	10	64
IV	1	4
V	7	12

## 4. Discussion

Most studies that have applied nuclear medicine to PJI have focused on the diagnostic efficacy of a single radionuclide or compared several radionuclides; few have compared or combined radionuclides with other laboratory tests (24, 25). Chen et al. (24) reported the combination of ^18^F-FDG and CRP in two-stage reconstruction; in their report, many patients had concomitant diseases such as percutaneous nephrostomy, cervical cancer, hemodialysis, and pyelonephritis and chronic diseases were common among older patients, which could lead to an elevation of CRP and confound the final diagnosis. ^18^F-FDG PET/CT was performed in these patients. Infection was defined as no significant increase in ^18^F-FDG uptake between the bone and antibiotic-loaded cement spacer; the infection was controlled and reimplantation was performed with satisfactory results. PJI is often accompanied by pneumonia, skin disease, and even malignancy. In these cases, serologic results are often unstable and ^18^F-FDG PET/CT is more useful for determining the etiology of the infection owing to its sensitivity and the fact that it allows visualization of the hip joint area, thereby facilitating diagnosis. In our study there was no significant correlation between SUV_max_ and any serologic parameter, which is also an advantage of using ^18^F-FDG PET/CT for PJI diagnosis as it can serve as an independent and reliable method to exclude potential infection.

In 2011, the MSIS proposed new diagnostic criteria for PJI by combining multiple examinations (26); these were modified in 2018 as evidence-based and validated criteria (27). Although PJI diagnostic criteria continue to evolve, research on the application of ^18^F-FDG to PJI has apparently stalled (14, 28), possibly because of the problem of non-specific ^18^F-FDG uptake. In one study, ^18^F-FDG uptake returned to normal after 3 months in fracture fixation patients (29). However, as soft tissue coverage of the hip joint was obviously greater than that of limb bone, the authors also performed ^18^F-FDG PET/CT in nine volunteers at 3, 6, and 12 months post hip replacement to assess the performance of ^18^F-FDG in that condition (4). They found moderate uptake at the head/neck of the prosthesis at 12 months, and concluded as did other investigators (15, 17, 30) that the uptake was non-specific and therefore not clinically meaningful.

In our study, there were four cases with an interval of <1 months between arthroplasty and PET/CT. The four cases showed diffuse uptake, but here, the intensity and range were not as substantial and extensive as would be expected. Although there is no information on the duration of soft tissue remodeling in hip arthroplasty, we think it unlikely that ^18^F-FDG reflects such remodeling.

Among cases with an arthroplasty-to-PET/CT interval >1 year, some did not have uptake, while others had diffuse but low-intensity uptake a few years later. In our previous animal study (31), we frequently observed non-specific uptake of ^18^F-FDG in muscle. Blood vessels and muscles were also visible in the images analyzed in the present work; this could account for the poor specificity of ^18^F-FDG for detecting PJI.

Another interesting finding was that among patients with apparent wear, some showed no uptake while others had high-intensity uptake around the joint cavity. Because of the retrospective nature of this study, patients were not evaluated for sensation or motion; as such, it is unclear whether this phenomenon indicated a soft tissue problem such as an inflammatory pseudotumor or foreign body reaction, which warrants further investigation.

We also found that ^18^F-FDG PET/CT had the potential for dynamic monitoring of the hip joint post arthroplasty. While none of the patients who underwent two PET/CT scans had specific findings, the performance of ^18^F-FDG PET/CT images of the two patients who had three scans were impressive. One patient underwent total hip arthroplasty during chemotherapy and ^18^F-FDG PET/CT was performed at 6, 18, and 31 months postoperatively. The range and intensity of uptake decreased gradually and at the last follow-up, sensation in the hip and mobility were satisfactory. Another patient with a tumor prosthesis underwent ^18^F-FDG PET/CT at 1, 5, and 12 months. Widespread low-intensity uptake was initially observed but the uptake became increasingly concentrated and intense over time. At the last follow-up, hip motion was acceptable but the patient experienced obvious discomfort during activities.

In addition to diagnosis, ^18^F-FDG PET/CT plays an important role in evaluating therapeutic response monitoring. With advances in PJI therapy and the possibility of non-surgical treatment of aseptic loosening, ^18^F-FDG PET/CT has potential application in the monitoring of clinical outcomes.

There are several limitations in this article. First, the number of images was relatively small, which would reduce the credibility of the analysis. Second, only non-infected patients were included and the performance of ^18^F-FDG PET/CT in PJI patients was not studied. Third, beacause the type of implants could not be determined (such as cemented or uncemented), the influence of prosthesis styles on ^18^F-FDG PET/CT could not be analyzed.

## 5. Conclusion

A review of non-infected hip arthroplasty cases examined by ^18^F-FDG PET/CT at our center revealed the following: (1) SUV_max_ can provide information that is useful for the diagnosis of PJI independent of serologic tests; (2) blood vessels and muscles can affect ^18^F-FDG uptake patterns in PJI; (3) ^18^F-FDG uptake was not as widespread as expected during soft tissue remodeling; (4) among patients with obvious wear, some did not have uptake while others had apparent uptake; and (5) ^18^F-FDG PET/CT is a promising tool for the dynamic observation of the hip post arthroplasty, with the advantages of high sensitivity and detailed visualization, and can be used in patients with unclear diagnosis. However, the specificity of ^18^F-FDG is not ideal and a more specific radionuclide is warranted for the application of PET/CT to PJI.

## Data availability statement

The raw data supporting the conclusions of this article will be made available by the authors, without undue reservation.

## Ethics statement

Ethical approval was not provided for this study on human participants because of the retrospective nature of this study, a waiver was granted by the Institutional Review Board of Chinese People’s Liberation Army General Hospital and no additional informed consent was required. The patients/participants provided their written informed consent to participate in this study. Written informed consent was obtained from the individual(s) for the publication of any potentially identifiable images or data included in this article.

## Author contributions

YQW, LY, and YD wrote the original draft. HL and YS ran the data curation and formal analysis. YC, QL, and YMW used the software. XM and SY supervised and designed the project. YZ and JT supervised, reviewed, and edited the draft. All authors contributed to the article and approved the submitted version.
